# Leishmaniasis Panamensis Masquerading as Myiasis and Sporotrichosis: A Clinical Pitfall

**DOI:** 10.1155/2015/949670

**Published:** 2015-08-30

**Authors:** Peter G. Pavlidakey, Thy Huynh, Kristopher Michael McKay, Naveed Sami

**Affiliations:** ^1^Department of Dermatology, University of Alabama at Birmingham, Birmingham, AL 35294, USA; ^2^Division of General Pediatrics, Children's Hospital Los Angeles, Los Angeles, CA 90027, USA

## Abstract

We report a case of cutaneous leishmaniasis panamensis in nonendemic Costa Rica. A 19-year-old female presented with nonhealing, unilateral eruption of erythematous papules with superficial central ulceration in a sporotrichoid pattern on right upper arm and back. Given the clinical picture and geographic locale, the patient was initially diagnosed with myiasis or human botfly infestation; however, the sporotrichoid pattern of the bites is an unlikely finding in myiasis. Peripheral blood smear, Giemsa stain, and polymerase chain reaction (PCR) were consistent for *Leishmania* spp. Ulceration resolved with 20-day course of IV sodium stibogluconate.

## 1. Introduction

Leishmaniasis is a parasitic infection caused by genus* Leishmania* spp. and transmitted by infected sandfly. Cutaneous leishmaniasis is categorized based on geographic distribution and divided into Old World versus New World. The regions of distribution ranges from Europe, Middle East, southwest Asia, and Africa to South America [1–3].

We present a case of cutaneous leishmaniasis panamensis; given the clinical picture and geographic locale, the patient was initially diagnosed with myiasis or human botfly infestation; however, the sporotrichoid pattern of the bites is an unlikely finding in myiasis.

## 2. Case Presentation

A previously healthy 19-year-old Caucasian female presented with a 4-week history of multiple nonhealing skin lesions after recent travel to the Costa Rican rain forests. A few days after returning to the United States, she noticed raised, red lesions on her right arm and back. The lesions began as painless, erythematous papules and progressed to superficial central ulceration with serous discharge. The patient did not use insect repellent and reported extensive insect bites that healed during the 2-week trip.

**Figure 1:**
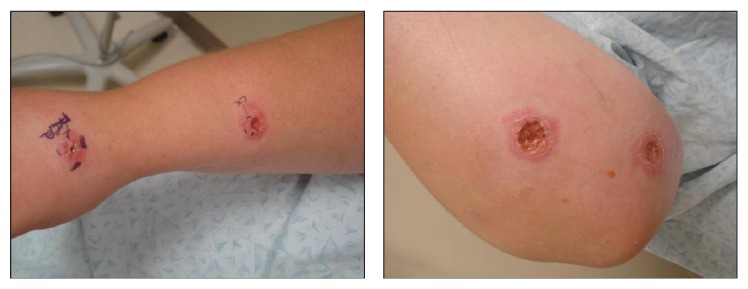
Patient with ulcerated, erythematous papules in sporotrichoid distribution on right upper arm.

Prior to being referred to dermatology, she was treated for myiasis or human botfly infestation with petroleum jelly occlusive wrapping and subsequently with oral trimethoprim-sulfamethoxazole without resolution. She presented with multiple painful 2-3 cm erythematous papules with elevated borders and central ulceration in a linear pattern on right forearm, upper arm, and upper back (Figures 1(a)-1(b)). No botfly larvae were appreciated or extracted during the physical exam.

The differential diagnoses included sporotrichosis, cutaneous leishmaniasis, Mycobacterial infection, cutaneous histoplasmosis, and cutaneous blastomycosis. Biopsies taken from the right upper arm were sent for pathology, tissue culture, and polymerase chain reaction (PCR). Findings were consistent for leishmaniasis panamensis and confirmed by Center of Disease Control (CDC). Baseline laboratory tests including complete blood count (CBC), comprehensive metabolic panel (CMP), amylase, and lipase were conducted prior to treatment and weekly thereafter. Lab workup and EKG were normal; pregnancy test was negative.

**Figure 2:**
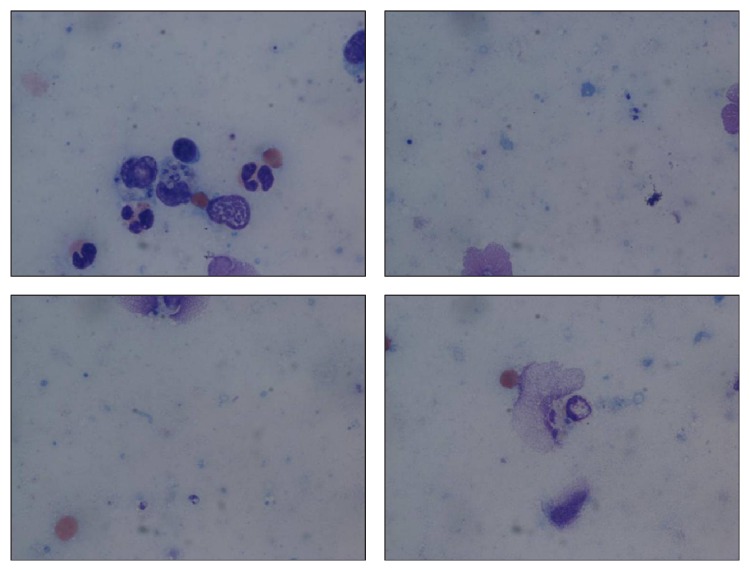
H&E smear: intact macrophages filled with* Leishmania* amastigotes also with visible nucleus and kinetoplast.

**Figure 3:**
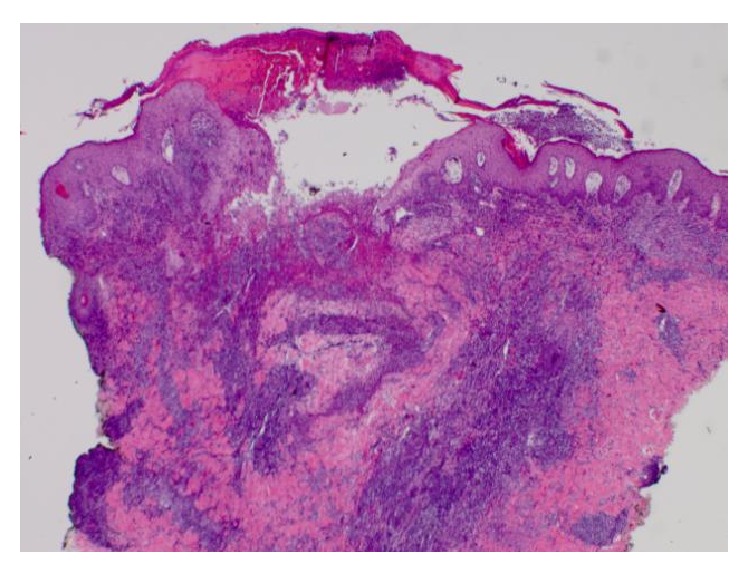
Low-power H&E: ulcerated papule with mixed superficial and also deep inflammatory infiltrate.

**Figure 4:**
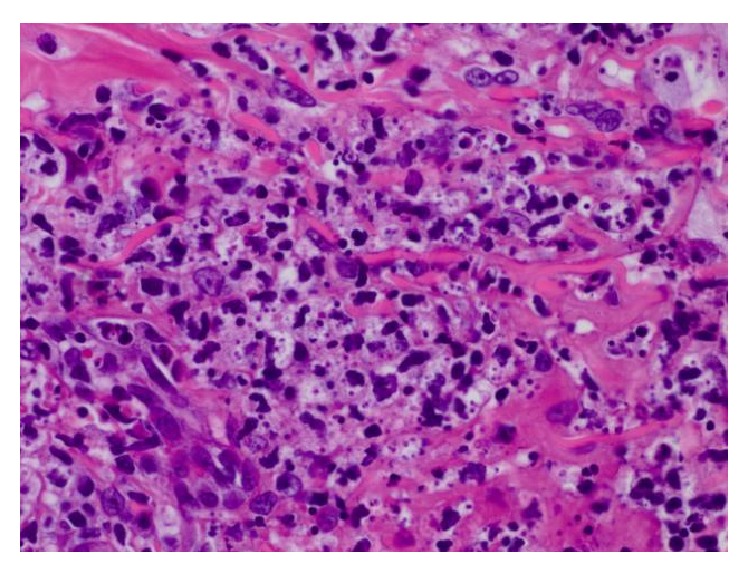
High-power H&E: parasitized histiocytes with amastigotes present.

**Figure 5:**
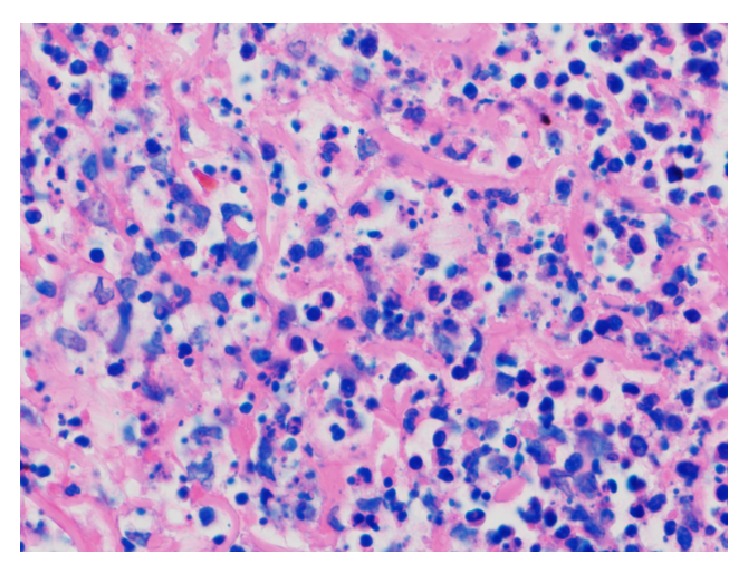
High-power Giemsa: parasitized histiocytes with diffuse staining of* Leishmania* amastigotes.

Histologically, the right arm biopsy showed intact macrophages filled with* Leishmania* amastigotes with visible nucleus and kinetoplast was demonstrated on the smear section (Figure 2). Peripheral blood smear showed normocytic red blood cells with normal morphology and rarely a few elliptocytes and polychromatic cells. Low-power hematoxylin and eosin (H&E) stain of ulcerated papule revealed mixed superficial and deep infiltrate (Figure 3). Both high-power H&E and Giemsa stain revealed parasitized histiocytes and diffuse staining of amastigotes consistent with* Leishmania* species (Figures 4 and 5). Gomori methenamine silver stain was negative. PCR was positive for* Leishmania* DNA.

She was treated with intravenous (IV) sodium stibogluconate 2000 mg daily for 20 days with resolution of ulceration. During which time she developed fatigue, headache, and myalgia during final dose of pentostam. Patient was asymptomatic although weekly EKG revealed QT prolongation that gradually shortened during course of treatment. Overall, she had good clinical response, minimal toxicity, and residual scarring at bite sites after treatment with sodium stibogluconate.

## 3. Discussion

Leishmaniasis is a parasitic infection caused by genus* Leishmania* spp. (Family Trypanosomatidae) and transmitted by infected Phlebotomine sandfly [1]. Cutaneous leishmaniasis (CL) is categorized based on geographic distribution and divided into Old World versus New World CL. Old World CL is caused by five species (*L. infantum*,* L. tropica*,* L. major*,* L. aethiopica*, and* L. donovani*) and affects regions of Europe (particularly Mediterranean Basin), Middle East, southwest Asia, and Africa [1–3]. In contrast, New World CL is transmitted by multiple species of both* Leishmania* and* Viannia* subgenera (*L. mexicana*,* L. (V.) braziliensis*,* L. (V.) panamensis*,* L. peruviana*, etc.) and remains a prominent zoonotic disease in South America, particularly endemic to Colombia, Brazil, and Peru [1, 3]. The risk of mucosal involvement varies with the various subtypes; however, it has been reported to occur with* L. panamensis *which necessitates the need for treatment.

Identification of* Leishmania* parasites or DNA via tissue culture, PCR, or isoenzymatic electrophoresis provides definitive diagnosis of CL [3–5]. Current gold standard for treatment is pentavalent antimonials (meglumine antimoniate and sodium stibogluconate) with notable side effects that include cardiac toxicity like prolonged QT interval and also hepatotoxicity [6]. Other options for therapy include monotherapy or various combination therapies with pentamidine, miltefosine, ketoconazole, azithromycin, or fluconazole [3, 6].

This case illustrates the importance of considering NWCL on the differential diagnosis even in nonendemic locations, along with keeping a wide differential even for somewhat prototypical lesions, as many infectious agents can present in a similar fashion.
